# A novel angular dependency model for MatriXX response and its application to true composite dose verification for IMRT plans

**DOI:** 10.1002/acm2.13405

**Published:** 2021-08-28

**Authors:** Yin Zhou, Jiugao Sang, Haibo Chen, Meng Zhu, He Wang, Shuwei Zhai, Lina Lu, Hui Liu, Zhengfei Zhu, Zhouguang Hui, Jianrong Dai, Jian Huan

**Affiliations:** ^1^ Evidance Medical Technologies Inc. Suzhou China; ^2^ Department of Radiation Oncology Rudong County People's Hospital Rudong China; ^3^ Department of Radiation Oncology The Affiliated Suzhou Science and Technology Town Hospital of Nanjing Medical University Suzhou China; ^4^ Homology Medical Technologies Inc. Ningbo China; ^5^ Department of Radiation Oncology State Key Laboratory of Oncology in South China Sun Yat‐sen University Cancer Center Guangzhou China; ^6^ Department of Radiation Oncology Fudan University Shanghai Cancer Center Shanghai China; ^7^ Department of Radiation Oncology National Cancer Center/Cancer Hospital Chinese Academy of Medical Sciences and Peking Union Medical College Beijing China

**Keywords:** angular response, IMRT, MatriXX, mechanism of angular dependency, patient specific QA, radiotherapy, true composite dose verification

## Abstract

**Purpose:**

This paper proposes a model for the angular dependency of MatriXX response and investigates whether MatriXX, with the angular‐model‐based approach can be applied to true composite dose verification for IMRT plans.

**Method:**

This model attributes the angular dependence of MatriXX response to dynamical translation of its effective measurement plane (EMP) due to the change of beam angle. Considering this mechanism, true composite dose verifications for IMRT plans specified in AAPM TG 119 report using both MatriXX and Gafchromic EBT3 films were undertook and compared to validate the applicability of MatriXX for patient specific QA of composite beam IMRT plans. Dose verifications using MatriXX with and without angular‐model‐based approach were performed.

**Results:**

MatriXX with angular‐model‐based approach achieved gamma passing rates with 3%/3 mm and 3%/2 mm criteria better than 98.3% and 98.1% respectively for true composite dose verification of plans in AAPM TG 119 report. The 3%/3 mm and 3%/2 mm gamma passing rates using MatriXX without angular‐model‐based approach ranged from 85.8% to 98.2% and from 81.3% to 96.5%, respectively. The *p*‐values from the single sided paired *t*‐test indicated no statistical difference between the passing rates from MatriXX with angular‐model‐based approach and from films, and significant difference between the passing rates from uncorrected MatriXX and from films.

**Conclusion:**

The proposed model for angular dependent MatriXX response is necessary and effective. Dose verification using MatriXX with angular‐model‐based approach is acceptable for true composite beam IMRT plans with required accuracy to simplify patient specific QA.

## 1 INTRODUCTION

Two‐dimensional (2D) ionization chamber array I'mRT MatriXX (IBA Dosimetry, Schwarzenbruck, Germany)^1^, ^2^ has been widely used and characterized for dose verification of IMRT^3^ from the perspective of efficient and reliable quality assurance (QA).^4^, ^5^, ^6^ Herzen et al^4^ performed extensive evaluations of the implementation of MatriXX in clinical routine including dependence on dose, energy and time, as well as determination of the effective point of measurement (EPOM) for perpendicular beam incidence. However, MatriXX is designed^2^ to measure doses for beams that are vertical to its front surface, not for beams in true composite dose verification which are set at their original planned angles. The angular dependence of MatriXX would affect the measurement accuracy in true composite dose verification of IMRT plans. Several MatriXX detectors have been tested, and rather large dose bias (up to 8%–11%) was observed for the non‐zero‐degree beams.^7^, ^8^ Hence, the true composite dose verification^9^ was normally performed using dosimetric detectors without angular dependence of response, such as Gafchromic EBT3 films.^10^, ^11^ Nevertheless, the tedious calibration of films limits their uses for routine QA. For that reason, seeking affordable alternatives appears attractive to daily practice. MatriXX could be an efficient and economically viable option for true composite dose verification for patient‐specific IMRT QA if its angular response could be accurately modeled and incorporated into the dose verification process.

Related work addressing the above problem has been performed by several authors. Dobler et al^12^ reported the result of MatriXX for composite beam IMRT plan verification without the consideration of MatriXX angular dependence and achieved the gamma pass rates ranging from 77.2% to 99.6%, while 12%–18% of testing plans failed the 95% passing rate evaluation under the 3%/3 mm criteria. Wolfsberger el al^7^ assumed that the angular correction factor (CF) is constant within the detector plane for each angle and measured the CFs of the MatriXX in the reference phantom with an independent detector which does not have any significant angular dependencies. Shimohigashi et al^13^ considered the off‐axis dependence of CF in one dimension, and calculated CFs using MatriXX measured dose and the dose calculated by the Monte Carlo (MC) algorithm. Boggula et al^14^ and Robert et al^8^ calculated CFs using MatriXX measured dose and treatment planning system (TPS)‐calculated dose to investigate the angular dependence of all the pixel chamber detectors. While the former used the CFs for VMAT QA, the later applied them for the composite dose verification of IMRT. However, all of the above publications adopted some phenomenological forms of the CF method, and none of them dealt with the mechanism for the angular dependency of MatriXX response. The current study is therefore to propose such a mechanism and its associated novel model, and to investigate whether, incorporating this mechanism, MatriXX could be applied for the true composite dose verification of IMRT plans.

In this study, angular dependency of MatriXX response is explicitly explained by the dynamic displacement of effective measurement plane of MatriXX due to the change of beam angle. Based on this mechanism, three assumptions are proposed to formulate the novel Dynamical Effective Measurement Plane (DEMP) model of MatriXX in Section 2.5. Calibration procedure for the determination of DEMP model parameters is described in Section 2.6, along with the application of DEMP model in corrected composite dose calculation for the MatriXX response in Section 2.7. Testing plans for model verification are subsequently described in Section 2.8 and evaluation methods in Section 2.9. Hereafter, evaluation results are presented in Section 3 and finally discussion and conclusion are made in Sections 4 and 5.

## 2 MATERIALS AND METHODS

### 2.1 Linear accelerator

The measurements were performed on a Trilogy linear accelerator (Varian Medical Systems, Palo Alto, CA, USA) with 6 MV photons and 120‐leaf Millennium MLC. Interdigitation was allowed. The maximum field size of 40 cm × 40 cm was defined by the secondary collimator jaws. The minimum gantry rotation scale was 0.1°. The accelerator was set up for step‐and‐shoot IMRT.

### 2.2 Dosimetric detectors

**FIGURE 1:**
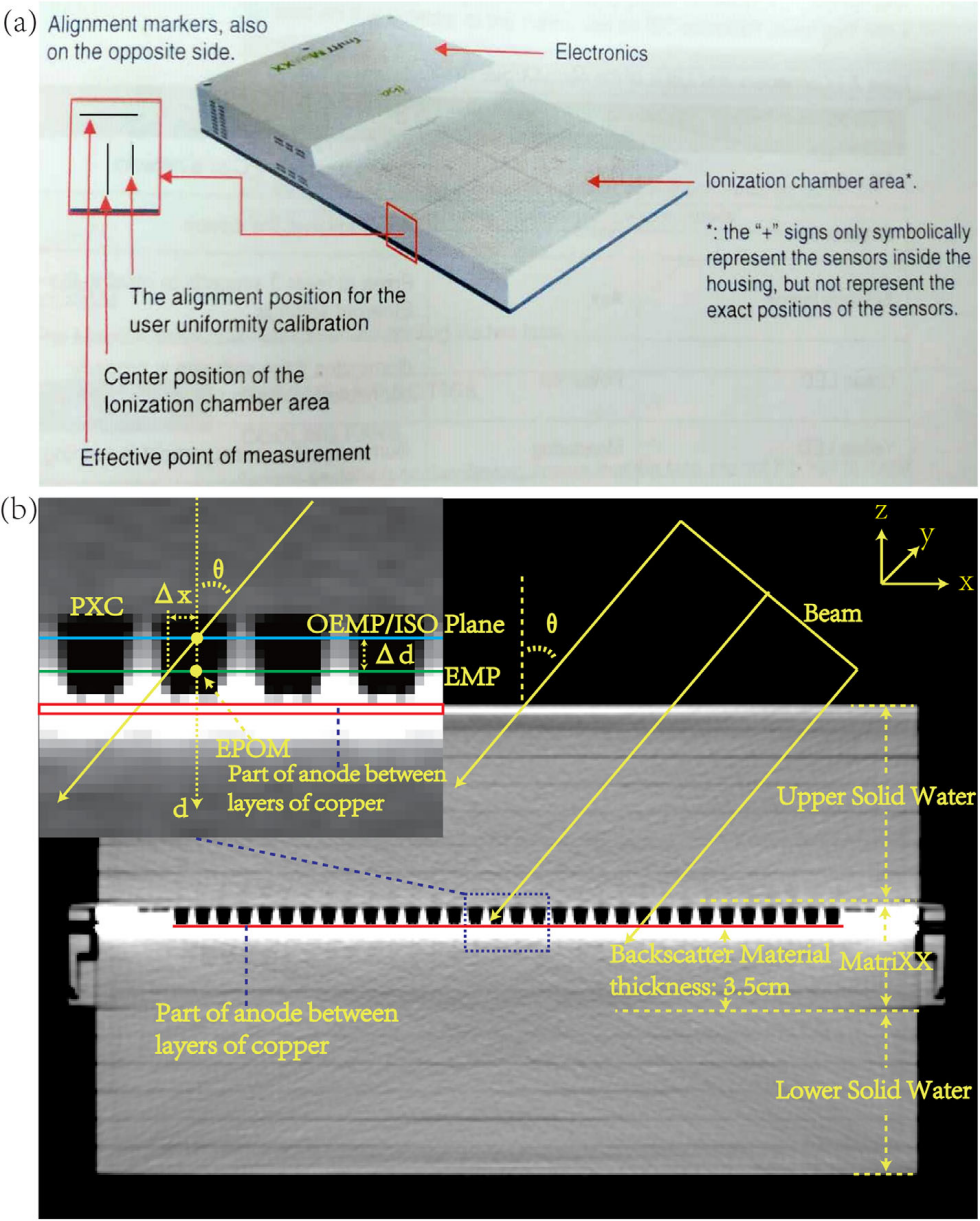
(a) The photo of two dimensional ionization chamber array MatriXX. (b) CT of MatriXX and the illustration of the dynamic effective measurement plane for MatriXX in the enlarged window

The 2D ionization chamber array MatriXX Evolution^1^, ^2^ was used for the true composite dose verification (Figure 1a). The MatriXX consists of 1020 air‐vented pixel ionization chambers (PXC) arranged in a square of 24.4 cm × 24.4 cm with a center‐to‐center distance of 7.62 mm. The chamber size is 4.5 mm for diameter and 5 mm for height, and the active volume is 0.08 cm^3^. The absorber material on top is 3‐mm thick and made of ABS Tecaran.^1^ The effective point of measurement for each PXC is at 3.5 mm below the surface and marked at both sides of MatriXX, as shown in Figure 1a. The deviation from linearity is ≤1% for dose ≥ 0.02 Gy.^1^, ^4^ MatriXX Evolution, which is an upgraded version of MatriXX, has been developed for composite dose verification by adding an inclinometer to record the gantry angle. The angular dependence of MatriXX Evolution was improved by replacing the metal screws on the body with plastic screws and adding a scatterer under the detectors. In this study, however, the inclinometer of MatriXX Evolution was not enabled. Figure 1b shows the CT scan of the MatriXX Evolution. From this figure, the thickness of the backscatter material inside the MatriXX was measured to be 3.5 cm, which is not explicitly indicated in the MatriXX manual.^1^ myQA platform from IBA was used to read the data from MatriXX and export the data to other patient specific QA software for further analysis. Before patient specific dose verification can be carried out, both the uniformity calibration and absolute calibration were performed for MatriXX.

Gafchromic EBT3 films (Ashland, Bridgewater, NJ, USA) were used as the self‐developing detector for high resolution dose measurement.^15^ For each film, the exposition duration for radiation is 200 MU. The film reading process was composed of three steps: 1. lot calibration with film strips; 2. film scanning and reading; 3. film calibration and gamma evaluation. In step 1 and 2, Epson 800 was adopted as the film scanner, and the scanning for film strips and films were performed both prior to and after irradiation, using a resolution of 50 dpi, 48 bit full‐color, reflection mode and multichannel dosimetry (all RGB channels), with all color corrections in the scanner turned off. In step 3, www.radiochromic.com, an FDA‐approved online web application, was used as film calibration and analysis tool to analyze measured in‐plane dose distribution on the film,^16^, ^17^ and the functionality of lateral correction for the film was enabled in this software.

### 2.3 Phantom setup

The measurement setup for dose verification, including the water equivalent depths above and below the iso‐plane, complied with the specifications in AAPM TG 119 report^18^ for the four IMRT plans to evaluate. For MatriXX, the iso‐plane was set at the depth of the effective point of measurement marked on the side of MatriXX by the manufacturer. For EBT3 films, the iso‐plane was set at the position of film. Slabs of IBA solid water SP34,^1^ which were made of RW3 (PTW, Freiburg, Germany), were used as additional build‐up (upper solid water) and backscatter material (lower solid water).

### 2.4 Treatment planning system

The TPS Zeus Cloud TPS v1.0 (Homology Medical, Ningbo, China)^19^ with collapsed cone (CC) dose calculation algorithm was used in this study. A dual source model of the accelerator's treatment head was implemented in Zeus Cloud TPS according to the requirements of AAPM TG 53 report.^20^ The whole commissioning process of beam model and MLC model was supervised by a third‐party institution.^21^ A certificate for passing all the required tests in IAEA TECDOC 1540 and AAPM TG 119 was issued for Zeus Cloud TPS. Some of the dose verification results for the true composite dose using EBT3 films will also be presented in Section 3.2, confirming the acceptable dose calculation accuracy of Zeus Cloud TPS. To achieve a high resolution in the coronal plane which was later used for comparison to the measurement, dose distributions were calculated using a dose grid of 1 mm × 1 mm × 1 mm in TPS. The TPS retained the 16‐bit CT data after import.

### 2.5 Angular dependency model of MatriXX response: The displacement of the effective measurement plane

**FIGURE 2:**
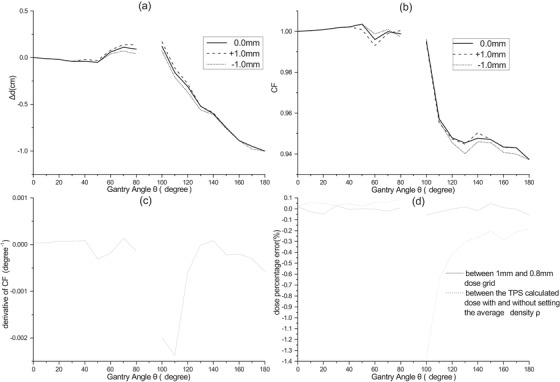
(a) The relation between depth offset Δd of the EMP of the MatriXX and the gantry angle and the sensitivity of Δd with respect to set up uncertainty. (b) Reproduce of the CF curve for the center PXC with DEMP and the sensitivity of the CF curve with respect to set up uncertainty. (c) Estimation of the derivative of the CF curve. (d) Pixel‐wise average dose percentage error for dose calculated with 1 mm dose gird and with 0.8 mm dose grid, and with and without the parmaeter *ρ*

The model is illustrated in Figures 1b and 2a and developed based on three assumptions:

***Assumption 1***. When the beam is set at 0°, the effective measurement plane (EMP) for MatriXX is located at 3.5 mm below its surface; when the beam is set at any other angle, the EMP is dynamically located somewhere different from this original EMP (OEMP) with an offset *Δd* (Figure 1b).
***Assumption 2***. The angular dependency of the MatriXX response is left‐right symmetric, that is, for the beam with gantry angle *θ*, and the beam with gantry angle 360° ‐ *θ*, MatriXX shares the same EMP.
***Assumption 3***. The thickness of part of the anode for each PXC within two thin layers of copper is 0.75 mm, and its effective density is ρ.

Herein, the EMP (Figure 1b) is defined as the plane containing the effective points of measurement (EPOM) (Figure 1a) of all the MatriXX pixel chambers, and the positive direction for the depth is defined as the direction pointing toward the bottom of the MatriXX. The original EMP (OEMP) is defined as the EMP of the MatriXX when the gantry angle is set at 0°. The unit of gantry angle *θ* is degree. Since there is a layer of high‐density material underneath the ionization chambers,^7^ the radiological path length of the radiation rays with gantry angle close to 90° and 270° is significantly longer than those with other angles. The assumption 3 is related to the fact that the region between copper layers in anode is thinner than 0.75 mm. However, since the CT resolution used in this study was 0.75 mm, an ROI of height 0.75 mm was delineated using a customized CT window level and width (WL = 1000, WW = 500) around the region of the highest density in CT of MatriXX. Since only part of this ROI consists of copper foils, and the rest of this ROI is made of glass fiber and epoxy, the ROI pixel's CT value would have been averaged by the CT scanner, possibly in some nonlinear way. Therefore, a parameter representing the nonlinear average density of this ROI is developed as ρ. This density is set into the delineated ROI by overwriting the CT value derived density (from CT‐to‐density table) and considered in the dose calculation.

In summary, the proposed angular dependency model of MatriXX response has a set of parameters *Δd*(*θ*), one per discretized gantry angle, and the parameter of ρ with its aforementioned meanings. For gantry angles other than the discretized angles, linear interpolation using *Δd of* two nearby discretized angles are employed. However, this interpolation may introduce large uncertainty in the angle interval of (80°, 100°) and (260°, 280°), where erratic fluctuation of *Δd*(*θ*) might occur as suggested by the CF values with large variation in the refs. 7 and 14. The essence of the model states that the angular dependency of the MatriXX response is caused by the dynamical displacement of its EPM as the beam angle varies around the 360°, with parameters *Δd*(*θ*) giving the relation between the depth offset of EMP and the beam angle. For any beam, the dose calculation to represent the MatriXX response should be performed on the EMP whose position is determined by the beam angle. Hereafter, we denote the proposed angular dependency model of MatriXX response as the DEMP model.

### 2.6 Determination of the DEMP model parameters

**FIGURE 3:**
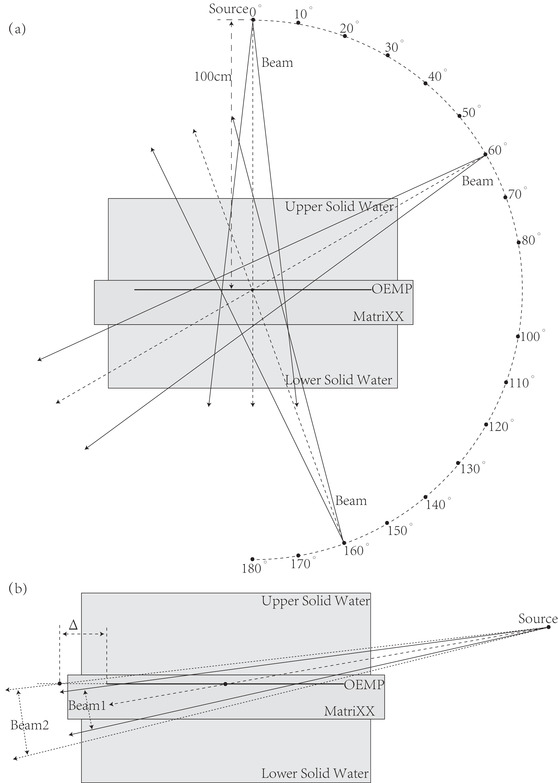
(a) The experimental setup for the DEMP model calibration. (b) Illustration of the irradiation area on the surface of the MatriXX. When the gantry is set close to 90, the beam 2 with larger aperture has an irradiation margin (marked by Δ) of the sensitive region of MatriXX

**FIGURE 4:**
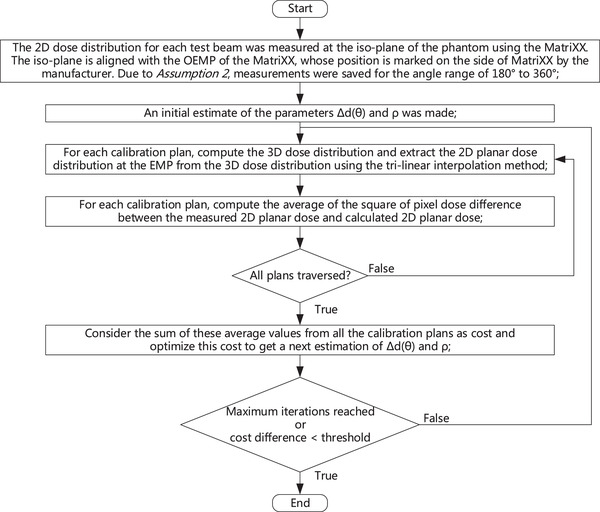
The workflow of the determination of DEMP model parameters

In order to apply the DEMP model in the composite dose calculation for the MatriXX response in clinical settings, the parameters of the DEMP model *Δd*(*θ*) and *ρ* must be determined based on the measurements of MatriXX angular response. In this study, the parameters of *Δd*(*θ*) were chosen at 19 angles ranging from 0° to 180° with an incremental angle of 10°. These parameters were then optimized based on a set of dose distributions measured from 19 single beam calibration plans corresponding to these angles. For each calibration plan, only one jaw‐collimated static 4 cm × 10 cm aperture was used for its single beam. The experimental setup of the calibration phantom is illustrated in Figure 3a. Slabs of IBA solid water SP34^1^ were used as additional build‐up and backscatter material, with respective heights of 7 cm and 8 cm. The distance between the source and OEMP of the MatriXX is set at 100 cm. The full procedure of the determination of DEMP model parameters in general is listed as following (Figure 4):

1. The 2D dose distribution for each test beam was measured at the iso‐plane of the phantom using the MatriXX. The iso‐plane is aligned with the OEMP of the MatriXX, whose position is marked on the side of MatriXX by the manufacturer. Due to *Assumption 2*, measurements were saved for the angle range of 180°–360°.
2. An initial estimate of the parameters *Δd*(*θ*) and *ρ* was made;
3. For each calibration plan, compute the 3D dose distribution and extract the 2D planar dose distribution at the EMP from the 3D dose distribution using the tri‐linear interpolation method.
4. For each calibration plan, compute the average of the square of pixel dose difference between the measured 2D planar dose and calculated 2D planar dose from step 3.
5. Consider the sum of these average values from all the calibration plans as cost and optimize this cost to get a next estimation of *Δd*(*θ*) and *ρ*.
6. Once the maximum number of optimization iterations was reached or the relative cost difference between last two iterations was smaller than a pre‐defined threshold, the procedure exited; otherwise, it would loop back to step 3 for further iterations of optimization.

In the above procedure, we employed Zeus Cloud TPS as the dose engine to compute the 3D dose distribution and Levenberg‐Marquardt method as the optimization algorithm^22^ to optimize the parameters of *Δd*(*θ*) and *ρ*. Herein, the x and y dimension of static beam aperture correspond to the moving direction of MLC and the direction perpendicular to it. The planar dose extraction method was implemented in the patient QA module of Zeus Cloud TPS. In more general circumstances, other commercial or in‐house TPSs are applicable for the per beam 3D dose calculation and the scripting function of those TPSs might be employed to implement the interpolation of *Δd*, the planar dose extraction procedure and the specification of *ρ* for the region of interest. Thus the above procedure for determining DEMP model parameters (*Δd*(*θ*) and *ρ*) could be transferred to other commercial TPSs smoothly. For each test beam, the 4 cm × 10 cm aperture was chosen, instead of normal 10 cm × 10 cm or even larger apertures. If the aperture width is greater than 4 cm, the sensitive area of the MatriXX would not contain the penumbra region of the dose distribution formed at the edge of the aperture, which might have large dose gradient and was of great significance for the accurate calibration of the angular dependency model, when the beam angle was close to 90°, for example, 80° used in our model calibration. This interpretation is further illustrated in Figure 3b, where the symbol Δ denotes the dose region of the sensitive area of the MatriXX. In order to confirm the acceptable error introduced in the interpolation of dose for the DEMP model calibration, pixel‐wise average percentage errors between dose distribution based on 1 mm dose grid and 0.8 mm dose grid for the 19 single beam calibration plans were calculated. The sensitivity of dose computation with respect to the average density *ρ* specified to the delineated ROI of the highest density in CT of MatriXX is also calculated for all the calibration plans by presenting the average percentage error between the TPS calculated dose with and without setting the average density ρ.

### 2.7 Application of the DEMP model in the corrected composite dose calculation for the MatriXX response

**FIGURE 5:**
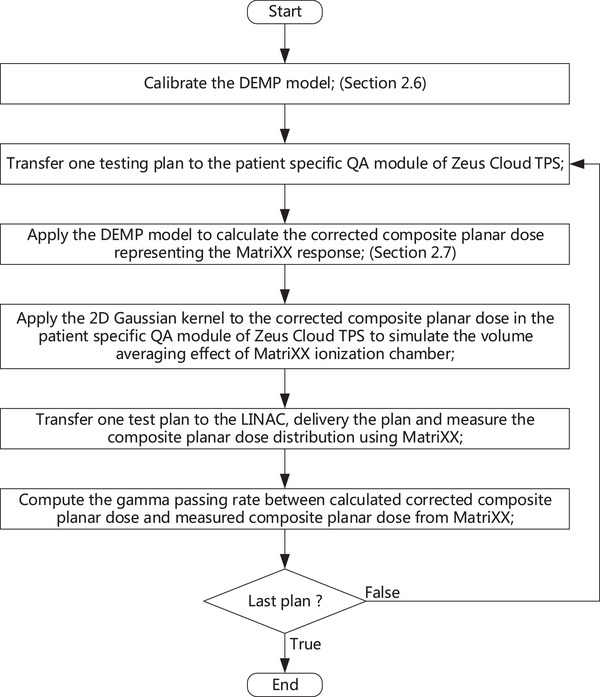
The workflow of composite dose verification for testing plans using the MatriXX with DEMP model

Once the parameters of the DEMP model were obtained, for a particular IMRT plan, the calculation of the corrected true composite dose to represent the MatriXX response could be performed (Figure 5). It followed as:

1. Transfer the IMRT plan to the patient QA module in Zeus Cloud TPS.
2. For each beam in the plan, 3D dose distribution was calculated by the patient QA module in Zeus Cloud TPS.
3. For each beam in the plan, extract the planar dose distribution at the EMP with depth offset of *Δd*(*θ*) from its 3D dose distribution using the tri‐linear interpolation method.
4. Sum up the planar dose distributions at the various EMPs from all the planned beams to obtain the final true composite planar dose distribution, representing the true composite response from the MatriXX.

Again, the patient QA module in Zeus TPS in the above procedure could be replaced by other commercial or in‐house TPSs and their scripting functions to perform 3D dose calculation and planar dose interpolation at a specified depth, in more general circumstances as described in Section 2.6. However, other patient‐specific QA softwares such as myQA platform or OmniPro I'mRT software are still not competent to this task since there is no functionality of extraction of planar dose distribution at an arbitrary depth from imported 3D dose yet.

### 2.8 Testing plans

To verify the efficacy of the DEMP model of the MatriXX angular response and its associated dose correcting methodology, eight open field single beam testing plans, one conformal plan, and four IMRT testing plans specified by AAPM TG 119 report^18^ were transferred to a CT study of the phantom specified in Section 2.3, recalculated and compared to measurements on the linear accelerator to simulate the true composite dose verification in patient QA. The eight single beam plans are jaw‐collimated with the field sizes of 4 cm × 4 cm and 5 cm × 3 cm. For each field size, four different beam angles are included: 0°, 50°, 130°, and 180°. The conformal plan is a seven‐beam MLC conformed plan designed on Zeus Cloud TPS for the case of Mock prostate in AAPM TG 119 report. The IMRT testing plans, including Test l1: Multitarget, Test l2: Mock prostate, Test l3: Mock head/neck and Test l4 C shape, were designed and optimized on Zeus Cloud TPS^19^ satisfying all the target dose requirements specified in AAPM TG 119 report. All the single beam and conformal plans, encompassed in this study to benchmark the accuracy of TPS modelling, could be regarded as special cases for true composite plans. To account for the treatment couch absorption, the original couch in simulation CT for any treatment plan was removed, and a calibrated treatment couch model was included in the CT scan for dose calculation and plan optimization.^23^, ^24^

### 2.9 Model verification

MatriXX and EBT3 films were used sequentially as the in‐plane dosimetric detector for the true composite irradiation. Measurements from the EBT3 films were treated as the baseline value to evaluate the performance of MatriXX with DEMP model for true composite dose verification. The workflow of composite dose verification for testing plans using the MatriXX with DEMP model is shown schematically in Figure 5. In the workflow, calculated composite planar dose to compare with the MatriXX response was corrected using the methodology presented in Section 2.7. Following the practice to represent the dose averaging effect of detector response function for the 2D ionisation chamber array like MatriXX with large pixel chamber,^25^ an additional convolution with a Gaussian kernel was applied in the patient QA module of Zeus TPS for the calculated planar dose distribution for its comparison with the measurement from MatriXX. Gamma evaluations were performed between the calculated dose and the measured dose.

True composite dose verifications using MatriXX without angular correction were also performed for the comparison purpose. Paired *t*‐tests for the gamma passing rates between measured and calculated dose, for any two of the three measurement modalities: MatriXX corrected with DEMP model, MatriXX without angular correction and EBT3 films, were performed to reveal the performance difference among these modalities. In each paired *t*‐test, for each measurement modality, four composite‐beam irradiations and totally six planar measurements were performed based on the four IMRT testing plans. Furthermore, to quantify the role of parameter *ρ*, dose verification of single beam testing plans using MatriXX with DEMP model but without parameter *ρ* was performed and compared with those from the full DEMP model. In addition to the gamma evaluation, 1D dose profiles and 2D dose discrepancy distributions were plotted.

Gamma evaluations^26^ in this study were performed both with 3% dose tolerance and 3 mm DTA recommended by AAPM TG 119 report^18^ and 3% dose tolerance and 2 mm DTA recommended by the most recent AAPM TG 218 report,^9^ across the whole pixel matrix at different depths from the phantom surface to investigate the potential and limitations of the mechanism. Measured planar data were linearly interpolated to 1 mm × 1 mm pixel size to be able to use a reasonably low distance to agreement which would otherwise be limited by the resolution of the measurement of 7.62 mm of MatriXX. As recommended by,^9^ only the region of interest with dose above the 10% of the maximum dose was involved in the passing rate computation to exclude the low dose region that has no or little clinical relevance but can significantly bias the analysis, and global normalization, also called Van Dyk percentage difference,^27^ was used for the computation of the relative dose difference with respect to the maximum dose in the region.^9^ All these gamma evaluations were performed in the patient QA module of Zeus TPS, while other alternatives such as myQA platform or OmniPro I'mRT software could be used too in normal clinical settings.

Furthermore, to compare with the CF curves defined in ref. 7, the effective CF curve was reproduced for the center PXC with DEMP. Here we apply the formula: CF = dose calculated at the center of the EMP/dose calculated at the center of the iso‐plane (OEMP), to simulate the CF defined in ref. 7. Different CF curves with simulated set up error in AP‐PA direction of 1 mm were calculated to estimate the sensitivity of CF curve to set up uncertainty. The derivatives of the CF curve were also calculated to reveal the sensitive of CF to gantry angle.

## 3 RESULTS

### 3.1 Model calibration and its parameters

**FIGURE 6:**
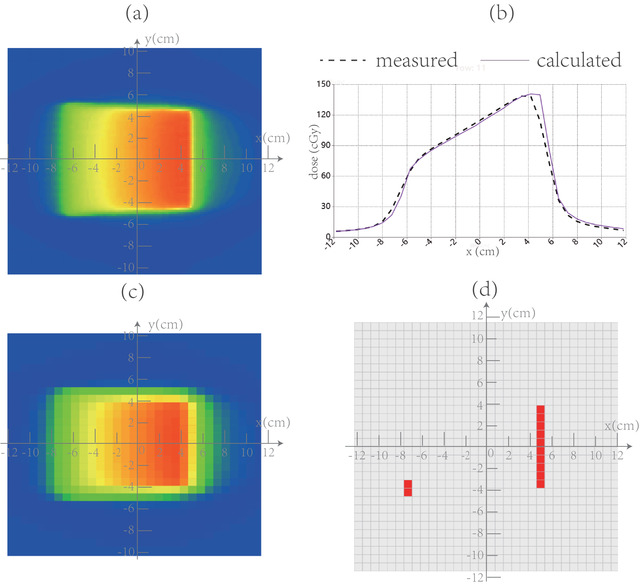
Comparison of (a) calculated (uncorrected) and (c) measured dose distributions, (b) dose profiles with horizontal coordinate representing x direction and (d) pixels failing the 3%/3 mm passing criterion for one of the single beam calibration plans when the gantry is set at 80°. The systematic lateral displacements of the measured dose profile, from that of the calculated dose profile uncorrected by the DEMP model, are quite obvious in (b). This displacement is also illustrated in Figure 1 as Δx

Figure 6 illustrates the calculated (uncorrected) and measured dose distributions, dose profiles and locations of dose discrepancy for one of the single beam test plans with the gantry angle at 70°. The systematic lateral displacements of the measured dose profile, from that of the calculated dose profile uncorrected by the DEMP model, are quite obvious in Figure 6b. This displacement is also illustrated in Figure 1 as *Δx*.

**FIGURE 7:**
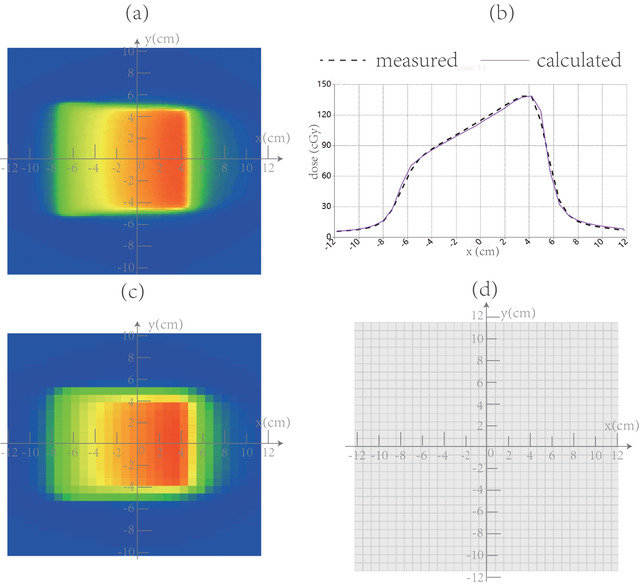
Comparison of (a) calculated (corrected) and (c) measured dose distributions, (b) dose profiles and (d) pixels failing the 3%/3 mm passing criterion for one of the single beam calibration plans when the gantry is set at 80°

Following the model calibration procedures listed in Section 2.7, the DEMP model parameters of *Δd*(*θ*) were determined and plotted in Figure 2a, and *ρ* = 3.5 g/cm^3^. For the sake of large potential uncertainty involved in the angle interval of (80°, 100°) and (260°, 280°), as indicated in Section 2.6, the *Δd*(*θ*) was not plotted for these intervals. Once the model is determined, Figure 7 illustrates the calculated (corrected) and measured dose distributions, dose profiles and locations of dose discrepancy for same plan as in Figure 6. It could be seen that there is no longer lateral displacement of the measured dose profile from that of the calculated one corrected by the DEMP model in Figure 7b.

Figure 2b shows that the reproduction of the CF curve for the center PXC with DEMP are analogous to that in ref. 7. In Figure 2a and b, the depth offset curve of the EMP and CF curve are both redrawn when the OEMP is translated by simulated set up offsets in AP‐PA direction by 1 mm. It could be seen that the differences between the redrawn curves are relatively small. In addition, Figure 2c reveals large fluctuations of the derivative of CF curve in the range of (60°, 80°) and (100°, 120°), with much large absolute value of derivatives in the range of (100°, 120°) than those in the range of (60°, 80°). Figure 2d displays the average percentage error between the interpolated dose on EMP with 1 mm dose grid and with 0.8 mm dose grid for the calibration plans. It is demonstrated that the average percentage errors are smaller than 0.05%, which are negligible for the 3%/3 mm and 2%/2 mm gamma passing rates of the dose verification. In this figure, it is also shown that the average percentage error between the TPS‐calculated dose with and without setting the average density ρ is negligible for the beams in the AP direction and greater than 1.3% when the angle angle is in the PA direction and close to 90°.

### 3.2 True composite dose verification

Table 1 gives the benchmark results of dose verification for the single beam and conformal plans, including the 3%/3‐mm and 3%/2‐mm gamma passing rates using MatriXX with and without the DEMP model, and using Gafchromic EBT3 films. It could be seen that MatriXX and Gafchromic EBT3 films respectively achieved gamma passing rates with 3%/3‐mm criterion better than 100.0% and 99.5%, and with 3%/2‐mm criterion better than 96.7% and 97.8% for dose verification for these testing plans. The 3%/3 mm and 3%/2 mm gamma passing rates without the consideration of angular dependency of EMP can be lowered to as much as 69.4% and 41.7%, respectively, in the single beam plan for the gantry angle of 130°. The 3%/3‐mm and 3%/2‐mm gamma passing rates with DEMP model but without the parameter *ρ* are slightly lower than those with the full DEMP model for both the single beam plans with beam angle of 130° and for one plan with beam angle of 180°.

**TABLE 1:** Dose verification of conformal and open field beams using MatriXX

		Single beam 4 cm*4 cm				Single beam 5 cm*3 cm				Conformal Plan
		0°	50°	130°	180°	0°	50°	130°	180°	
MatriXX with DEMP (%)	3%/3 mm	100.0	100.0	100.0	100.0	100.0	100.0	100.0	100.0	100.0
	3%/2 mm	100.0	98.5	97.3	96.7	100.0	100.0	97.2	100.0	99.8
MatriXX with DEMP but withou ρ (%)	3%/3 mm	100.0	100.0	100.0	100.0	100.0	100.0	100.0	100.0	100.0
	3%/2 mm	100.0	98.5	96.2	95.6	100.0	100.0	95.4	100.0	99.8
Uncorrected MatriXX (%)	3%/3 mm	100.0	100.0	76.7	83.3	100.0	100.0	69.4	83.3	95.1
	3%/2 mm	100.0	90.9	41.7	52.0	100.0	92.3	66.7	83.3	94.2
Film (%)	3%/3 mm	100.0	99.5	100.0	100.0	100.0	100.0	100.0	100.0	99.9
	3%/2 mm	100.0	97.8	99.9	100.0	100.0	100.0	100.0	100.0	99.2

Table 2 gives the results of the composite beam dose verification of the plans from Test l1 to Test l4, including the 3%/3 mm and 3%/2 mm gamma passing rates using MatriXX with and without the DEMP model, and using Gafchromic EBT3 films. It could be seen that MatriXX and Gafchromic EBT3 films respectively achieved gamma passing rates with 3%/3 mm criterion better than 98.3% and 99.1%, and with 3%/2 mm criterion better than 97.0% and 98.2% for dose verification for composite beam plans in AAPM TG 119 report. The 3%/3 mm and 3%/2 mm gamma passing rate without the consideration of angular dependency of EMP can be lowered to as much as 85.8% and 81.3% respectively. The *p*‐values from the single sided paired *t*‐test for the passing rates between MatriXX with DEMP based approach and films are 0.88 and 0.81 for 3%/3 mm and 3%/2 mm criteria, respectively, indicating no statistical difference between the QA results from these two measurement methods. The *p*‐values for the passing rates between uncorrected MatriXX and films, and between uncorrected MatriXX and MatriXX with DEMP based approach are all <0.05 for 3%/3 mm and 3%/2 mm criteria, respectively.

**TABLE 2:** Dose verification of true composite beam IMRT plans using MatriXX

		Multi Target	Prostate	Head/Neck		C Shape	
		ISO plane	ISO plane	ISO plane	4‐cm posterior	ISO plane	2.5‐cm anterior
MatriXX with DEMP (%)	3%/3 mm	100.0	100.0	99.0	100.0	99.1	98.3
	3%/2 mm	100.0	99.5	99.0	99.0	98.7	97.0
Uncorrected MatriXX (%)	3%/3 mm	96.5	88.0	95.8	85.8	98.2	92.1
	3%/2 mm	96.5	87.5	95.2	81.3	94.2	91.2
Film (%)	3%/3 mm	99.1	99.8	99.1	99.2	99.1	99.8
	3%/2 mm	99.0	99.7	98.7	98.2	98.7	99.6

**FIGURE 8:**
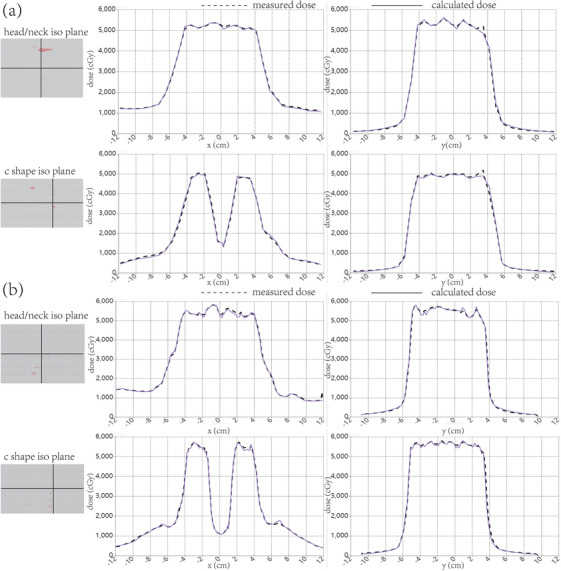
(a) The location of the gamma criterion failure and the comparison of profiles (position in 2D plane marked by the two bold lines) in x direction (parallel to the MLC moving direction) and y direction (perpendicular to the MLC moving direction) for two representative composite beam plans between the measurements from the MatriXX with DEMP model and the calculated dose. (b) The location of the gamma criterion failure and the comparison of profiles in x direction and y direction for two representative composite beam plans between the measurements from the EBT3 film and the calculated dose

Figure 8a,b shows the location of the gamma criteria failure (3%/3 mm) and the comparison of the calculated dose profiles in x and y directions for the two of the four composite beam plans with the measurements from MatriXX with DEMP based approach and Gafchromic EBT3 films, respectively. Excellent agreements can be seen, both between the calculated doses and the measurements from MatriXX with the DEMP model, and between the calculated doses and the measurements from EBT3 films.

## 4 DISCUSSION

EPOM is a well‐known concept in the dosimetry of ionization chamber, which is used to assign the measured absorbed dose in the chamber volume to a particular point in the undisturbed phantom.^28^ A judicious choice of EPOM could minimize the overall perturbation factors passing from ionization‐to‐air (the dose quantity measured) to dose to water.^29^ Since the response of PXCs is homogeneous after the uniformity calibration of MatriXX,^4^, ^7^ it is reasonable to speculate that the EPOMS of all the PXCs share the same depth. Accordingly, for 2D detector arrays such as MatriXX, a new concept called effective measurement plane (EMP) was proposed in this paper, which consisted of all the EPOMs of the PXCs. This study moved on to extend the concept of EMP at 0° gantry angle to all angles around the full 360° range. It was speculated that the EMP was angle dependent due to the anisotropic structure of the PXC.^2^, ^7^ We subsequently proposed, for the first time, a mechanism of the angular dependency of the MatriXX response by introducing the DEMP model. The angular dependency of MatriXX response was explained, in current study, by the dynamic displacement of the EMP due to the change of the beam angle. It is reasonable to consider the DEMP as a first‐order correction for the calculated dose when comparing the calculated dose with measurements from the MatriXX receiving radiation from different angles. Previous methods accounting for the angular dependency of MatriXX response mostly were based on some sort of CFs.^7^, ^8^, ^13^, ^14^ It should be noted that the DEMP model in this study is to calculate the angularly corrected composite planar dose, which is to be compared with the MatriXX response, using the TPS‐calculated dose distribution from each beam; while the conventional CF methods is to correct the MatriXX response from each beam and then synthesize them to match the TPS‐calculated dose on the measurement plane. The distinction between the two methods could be summarized as the conventional CF method corrects the MatriXX response, while the DEMP‐based approach corrects the TPS‐calculated planar dose. This distinction creates following three merits for the new approach.

Firstly, due to their phenomenological nature, CF methods normally require a large number of sampling points, for the rotational dimension of gantry angle and the off‐axis dimensions (2D) on the sensitive area of MatriXX, to make the correction sufficiently accurate. In this regard, the correction methods had evolved from using only CF for the center PXC for each angle,^7^ to using CFs of a line of PXCs,^13^ and to using CFs for all the PXCs on the whole 2D sensitive area^8^, ^14^ of MatriXX. The increase of the correction parameters demanded more time for MatriXX calibration and raised the possibility of overfitting. The novel mechanistic DEMP model developed in current study however had fewer parameters, which could greatly alleviate these problems, for example, the time it takes to estimate the corrected composite dose from Zeus Cloud TPS is within several seconds.

Secondly, the new approach could derive the beam angle information from treatment plan and saves the inclinometer, which is only available in an upgrade version of MatriXX – MatriXX Evolution. Although MatriXX Evolution is used in this study to enhance the angular dependence of the detector, its inclinometer is not yet enabled. This implies that the DEMP‐based approach is feasible even for radiotherapy centers with only MatriXX, which enlarges the application scope of the new approach over that of conventional CF methods.^7^, ^8^, ^13^, ^14^

Thirdly, in conventional CF methods, two different phantom setups are required for CF measurement in the model calibration process, one for Matrixx and the other for films, which would inevitably introduce uncertainties in the CF by the differences in density distributions of detectors. However, the new approach in this study needs only one detector of MatriXX and one phantom setup in the model calibration process which eliminates these density‐related uncertainties entirely.

After quantitative calibration of the DEMP model, the parameter values shown in Figure 2a indicates that the dynamic displacement of EMP could be as large as 1 cm, which could explain the deep valley around 180° (PA field) for the reproduced CF profiles in Figure 2b. The value of parameter *ρ* is 3.5 g/cm^3^, which was reasonable since it was the average density of the mixture of glass fiber (2.4 g/cm^3^), epoxy (1.0 g/cm^3^), and copper (8.96 g/cm^3^) in the anode. The asymmetry about 90° of the *Δd*(*θ*) agrees well with the asymmetric about 90° of the CF profiles. The reproduce of the CF profile in Figure 2b using the DEMP model showed analogous shape to those of previous works,^7^, ^8^, ^13^ verifying the efficacy of DEMP model. The fact that neither the depth offset curve of the EMP nor the CF curve is much influenced by the simulated set up error of 1 mm in Figure 2a,b demonstrated the robustness of the DEMP model to the set up uncertainties. Additionally, the derivatives of CF curve in Figure 2c revealed that the sensitivity of CF to the gantry angle is largest in the gantry angle range of (100°, 120°). It should also be noted from Figure 2d that the errors introduced by the linear interpolation in TPS's 1 mm dose grid are adequately small and acceptable for the calibration of the DEMP model. As indicated in Figure 2, neither *Δd*(*θ*) nor CF(*θ*) was plotted for the angle interval of (80°, 100°) and (260°, 280°), since the behavior of angular response in these angle range might be very different from those at other angles.^7^, ^13^ Ongoing work would be required to fully characterize the *Δd*(*θ*) in these angle ranges with finer sampling resolution. Figures 6 and 7 further demonstrated the effect of applying DEMP in correcting the dose calculation of oblique beam radiation for the comparison with the MatriXX response.

From Tables 1 and 2, it could be seen that the angular dependency of MatriXX response was essential when applying QA of single oblique beam plan, conformal plan, and true composite beam IMRT plan. After the adoption of DEMP model in true composite dose verification, the accuracy had been greatly improved, compared to the dose verification results from similar studies performed using conventional CF methods with MatriXX Evolution and its inclinometer.^7^, ^12^, ^13^ The promising results compared to Dobler's data^12^ might attribute to their exclusion of the angular dependency of MatriXX response. The discernible improvement over the result of Shimohigashi et al^13^ was possibly due to the inclusion of a more effective mechanistic model of the MatriXX angular response and a more accurate model of treatment couch in this study. It should be stressed that the difference in dose for PA versus AP fields could not be explained definitively in Wolfsberger et al.^7^ which only speculated the underlying cause to be some unknown effects occurring at the air‐high‐Z material interface for the PA beams. However, this study clearly manifested the role of the parameter *ρ* of the DEMP model in Table 1. The most significant improvement of the passing rates made by this paramete was for the PA fields with the beam angle of 130°, from which the length radiological path through the high desity region of anode beneath the PXC of Matrixx was relatively long. Thus, using the joint effects from the dynamical EMP, the high density of the anode, and the quantitative modeling of treatment couch, this study correctly predicted the dose difference for PA versus AP fields, which further confirmed the effectiveness of the overall methodology for the modeling of the angular response of MatriXX.

After the application of the DEMP model for MatriXX, it could also be seen from Table 2, and the *p* values of the paired *t*‐tests in Section 3.2 that true composite dose verification results from MatriXX were comparable to those from Gafchromic EBT3 films. However, there remained two caveats. First, the volume‐averaging effect caused by the relatively large sensitive volume size of PXC was unignorable. This study adopted a convolution correction method suggested by Herzen et al.^4^ and Poppe et al.^25^ that considers the response function of each detector. This method might enhance the verification results for dose distribution. Another problem of MatriXX was its low spatial resolution of 7.62 mm, which could be seen clearly in Figures 6c and 7c. When the dose was highly modulated between two ion chambers, the high frequency components of the dose between them would be smoothed out by the interpolation method used for dose comparison and gamma analysis, leading to information loss for MatriXX. The dose measured from MatriXX will be different from the value on the respective pixel of the calculated dose distribution, which results in dose deviations as seen in Figure 8a. Yet, such dose deviations could not be seen in Figure 8b for films. Special attention should also be paid to different shape of dose profiles in Figure 8a,b. This was due to the different CT image for phantom setup for different dosimeters.

Although the novel angular dependency model has been applied only in IMRT QA with MatriXX in current study, it would be interesting to note that the application could be extended to VMAT QA by replacing the beams in the IMRT plan, as described in Section 2.7, with the control points in VMAT plan, despite the number of control points in a VMAT plan is normally much larger than that of beams in a IMRT plan. Counter‐intuitively, a previous work also showed a viable result using MatriXX without any correction method for the composite dose verification of VMAT.^30^ This could be attributed to two points: 1. The gamma criteria used there are 3%/4 mm rather than 3%/3 mm or 2%/2 mm used in other works. 2. The angular dependence of MatriXX for VMAT QA might be compensated by irradiation from multiple gantry angles. The second point also highlighted a potentially more critical role played by the angular correction method for IMRT QA with MatriXX than for VMAT QA, since the facts of fewer beams and more highly modulated nature of IMRT elevated the degree of asymmetry of the angular distribution of radiation fluence so as to amplify the overall effect of angular dependency of MatriXX response. Following this line of reasoning, the angular correction method for patient specific QA with MatriXX could also be potentially applied to other advanced radiotherapy technologies, such as Helical TomoTherapy (HT), which employ highly asymmetric angular distribution of radiation fluence. However, it had been pointed out by Deshpande et al^31^ that in the HT setting, the conventional CF based angular correction for MatriXX Evolution cannot be applied as the dosimetry system relies on inclinometer input for obtaining the instantaneous gantry position information and the inclinometer cannot be attached to an HT unit due to the lack of gantry head access. On the contrary, as indicated above, no inclinometer is required for the DEMP model‐based approach, implying the possible improvement of the performance of MatriXX in detecting Helical TomoTherapy treatment delivery errors^31^ by using the angular correction method proposed in this study.

Despite the merits of the new approach, one notable concern about the practality of the DEMP‐based approach is whether the uncertainties in dose calculation in TPS would affect the accuracy of the DEMP model calibration. Catering for this concern, it is recommended that the TPS used for DEMP‐based correction approach be commissioned sufficiently through both the per beam and true composite dose verifications using Gafchromic EBT3 films to ensure that the uncertainties in TPS, especially the angular dependent uncertainties, are within the acceptable range and would not deviate unacceptably the results of dose verification using MatriXX with DEMP‐based approach in patient specific QA. Fortunately, these machine and TPS QA tasks are required only once for a relatively long period of time. Considering the all merits of the novel DEMP‐based approach indicated above, these extra QA efforts would be worthwhile.

## 5 CONCLUSION

The novel DEMP model proposed in current study for the angular dependency of MatriXX response is effective and accurate. It provides a more systematic way for the correction of angular response of MatriXX than the traditional CFs. It greatly shortens the calibration time and enlarges the application domain of the angular correction method by eliminating the requirement for the inclinometer to get the real‐time gantry angle information from the output of dosimeter. It is confirmed that the true composite dose verification process incorporating this model using MatriXX is acceptable with gamma passing rates better than 98.3% under the 3%/3 mm criterion and 98.1% under 3%/2 mm criterion for IMRT plans, which are comparable to those achieved by Gafchromic EBT3 films. MatriXX combined with the DEMP‐based approach could help to simplify patient‐specific QA with lower cost and higher efficiency in fixed‐gantry intensity modulated radiation therapy.

## AUTHOR CONTRIBUTIONS

Yin Zhou, Jiugao Sang and Jian Huan created the idea and designed the project scheme. Yin Zhou, Meng Zhu and He Wang designed and implemented the algorithm. Haibo Chen, Shuwei Zhai and Lina Lu performed the dose verification experiment. Hui Liu, Zhengfei Zhu, Zhouguang Hui and Jianrong Dai designed and optimized the testing plans and reviewed the paper. The authors also want to thank Dr. Ming Ji for his assistance in editing of this article.

## Data Availability

The data that support the findings of this study are available from the corresponding author upon reasonable request.
